# Three multiplex snapshot assays for SNP genotyping in candidate innate immune genes

**DOI:** 10.1186/1756-0500-6-54

**Published:** 2013-02-07

**Authors:** Lisa M Esteves, Sara M Bulhões, Maria J Brilhante, Luisa Mota-Vieira

**Affiliations:** 1 Molecular Genetics and Pathology Unit, Hospital of Divino Espírito Santo of Ponta Delgada, EPE, São Miguel Island, Azores, Portugal; 2 Instituto Gulbenkian de Ciência, Oeiras, Portugal; 3 Center for Biodiversity, Functional & Integrative Genomics (BIOFIG), Lisbon, Portugal

**Keywords:** Innate immune genes, Polymorphisms, SNaPshot multiplex PCR

## Abstract

**Background:**

Innate immune system is the first line of research when studying immune response to diverse infections and autoimmune/inflammatory diseases. This immune response has been reported to be genetically diverse, due to polymorphisms coded by different genes. For this reason, our purpose was to develop a multiplex assay that allows the genotyping of candidate single nucleotide polymorphisms (SNPs) in innate immune genes.

**Findings:**

We developed three multiplex PCR panels coupled with the minisequencing (SNaPshot) technique (multiplex PCR, multiplex primer extension, and capillary electrophoresis). The panels were tested in a sample set composed of 100 anonymous DNAs from healthy blood donors living in São Miguel Island (Azores, Portugal). Sixteen relevant SNPs among nine genes of the innate immune system – *IL1α*, *IL1β*, *IL6*, *IL10*, *IL12RB1*, *TLR2*, *TLR4*, *TLR9* and *CD14* – were genotyped and validated by direct sequencing, with the exception of one that was undetected by minisequencing. We suggest that these panels can be used in future studies for detection of risk gene variants in several populations and/or diseases.

**Conclusions:**

In summary, we propose a multiplex assay that is able to identify the most frequent candidate SNPs in innate immune genes, using a medium scale genotyping platform. The assays can be used to evaluate the risk gene variants in populations of various geographic origins.

## Findings

The innate immune system is involved in autoimmune/ inflammatory diseases, and is the first line of host defence against invading organisms. In part, most of these infective agents are successful in surviving, disseminating and infecting hosts, because of their ability to evade the innate immune system. Several studies suggested that, in human susceptibility to infection, host genetic makeup has a major role in innate immune response [1]. This susceptibility has been reported among infectious diseases such as tuberculosis [2], bacterial meningitis [3], leprosy [4] and hepatitis [5,6]. The genetic variation of innate immune response to pathogens usually involves a variety of polymorphisms among different genes, including those that encode toll-like receptors (TLRs) and interleukins (ILs).

The methods used to investigate genetic variation in *TLR*s and *IL*s are mostly singleplex PCR-based techniques, such as direct sequencing and real-time PCR. For small and medium-size laboratories, singleplex techniques became expensive and time-consuming when a large number of samples are evaluated. The alternatives to these methodologies, such as minisequencing assay – SNaPshot multiplex PCR –, have been successfully applied to support studies regarding oncogenic related genes [7-9], bacterial serotyping [10] and infectious diseases [11]. Moreover, the SNaPshot is a relatively simple and affordable method that allows more efficient and quick results when it is compared with singleplex techniques. In addition, it only requires the use of a thermal cycler and a capillary electrophoresis, which are equipments commonly available in molecular biology laboratories. In order to apply this technique to candidate genes studies associated with autoimmune and infectious diseases, we developed a multiplex assay designed to genotype 16 single nucleotide polymorphisms (SNPs) among nine genes of the innate immune system: *IL1α*, *IL1β*, *IL6*, *IL10*, *IL12RB1*, *TLR2*, *TLR4*, *TLR9* and *CD14*.

## Methods

### Selection of candidate innate immune system genes

Candidate genes and SNPs were selected considering their association to human autoimmune and infectious diseases. For this purpose, we used an updated and publically downloadable database – the Human Genome Epidemiology (HuGE) Literature Finder (http://www.hugenavigator.net) – that provides access to human genome epidemiology, including information on population prevalence of genetic variants, gene-disease associations, gene-gene and gene-environment interactions, and evaluation of genetic tests [12].

### Population samples and sequence analysis

The sample set was composed of DNA from 100 healthy blood donors living in São Miguel Island, obtained from the anonymized Azorean DNA bank located at the Hospital of Divino Espirito Santo of Ponta Delgada, EPE, of the same island [13]. This DNA bank was established after approval by the Ethics Committee for Health of the same Hospital, and follows the international ethical guidelines, which include informed consent, confidentiality, anonymity of personal data and abandonment option in case of expressed will.

From this sample set, 25 DNAs that evidence high SNP heterozygosity were selected to perform direct sequence analysis. Each 20 μl PCR reaction contained 100 ng of genomic DNA, 10 μM primers, 100 μM dNTPs (Promega), 25 nM MgCl_2_ (Qiagen), 1× Q-Solution (Qiagen), 1x buffer (Qiagen), 5 U of HotStart Taq (Qiagen) and sterile water. The PCR started with an enzyme activation step at 94°C for 15 min, then 40 cycles at 94°C for 1 min, 50°C for 1 min and 72°C for 1 min, followed by a final extension step at 72°C for 7 min. Amplification products were purified using the QIAquick PCR Purification Kit (Qiagen), according to the manufacturer’s instructions. Purified products were sequenced, using the same primers of the PCR amplification, with the BigDye Terminator v1.1 cycle sequencing kit (Applied Biosystems) under the following conditions: 1 μl ready reaction mix, 5 μl BigDye sequencing buffer, 3.2 pmol forward or reverse primer, 7 ng DNA and sterile water to a final reaction volume of 20 μl. Cycle sequencing was performed using initial denaturation step at 96°C for 1 min followed by 25 cycles at 96°C for 10 s, 50°C for 5 s and 60°C for 4 min in a GeneAmp® PCR System 2700 (Applied Biosystems). The sequencing products were purified with BigDye XTerminator® Purification Kit and separated by capillary electrophoresis in an automated sequencer (ABI 3130 Genetic Analizer, Applied Biosystems) with a 36 cm length capillary and POP-7™ polymer, according to the manufacturer’s instructions. Data were analyzed with Sequencing Analysis Software version 5.3.1 (Applied Biosystems). The alignment and edition of sequences were carried out using the Bioedit™ software version 7.0.0.

### Multiplex PCR primers and SNaPshot probes

The first step of this study was to design a multiplex PCR assay that includes all 16 SNPs among the nine genes of the innate immune system (Table 1). The key point in this step is the combination of primer pairs in a master mix. We used the PrimerPlex biosoft software [14]. This software is automatically linked to the SNP database (dbSNP, http://www.ncbi.nlm.nih.gov/projects/SNP/) and only requests the insert of SNP rs number. Using this information, the program picks the DNA sequences from the dbSNP and mixture them. The output of the program gives the best multiplex primers and probes pairs with the less secondary structures possible (Tables 2 and 3). For this reason, it was not possible to use less PCR fragments to capture the SNPs: bigger fragments comprising more SNPs would allow the formation of more secondary structures. In the multiplex primer extension panels, poly-T tails of different lengths were added to each probe at their 5’ ends, to separate extension products by size.

**Table 1:** Genetic information of the 16 selected SNPs

**Gene**			**SNP**		
**Name**	**Symbol**	**NM**	**dbSNP**	**Position**	**Variation**
*Interleukin 1 - alpha*	*IL1α*	000575.3	rs1800587	−889	C>T
*Interleukin 1 - beta*	*IL1β*	000576.2	rs16944	−511	A>G
*Interleukin 6*	*IL6*	000600.3	rs1800797	−596	A>G
			rs1800795	−174	C>G
*Interleukin 10*	*IL10*	000572.2	rs1800896	−1082	A>G
			rs1800872	−592	A>C
*Interleukin 12 receptor, beta 1*	*IL12RB1*	005535.1	rs11575934	+705	A>G
			rs401502	+1196	C>G
*Toll-like receptor 2*	*TLR2*	003264.3	rs4696480	−16933	A>T
			rs121917864	+2029	C>T
			rs5743708	+2259	A>G
*Toll-like receptor 4*	*TLR4*	138554.3	rs4986790	+896	A>G
			rs4986791	+1196	C>T
*Toll-like receptor 9*	*TLR9*	017442.3	rs187084	−1486	C>T
*Cluster of differentiation 14*	*CD14*	000591.3	rs2569190	−260	A>G
			rs2569191	−159	C>T

**Table 2:** Primer sequences used for multiplex PCR amplification panels

**Gene**	**SNP** **(rs)**	**Forward** **(5’→3’)**	**Reverse** **(5’→3’)**	**Amplicon size (bp)**	**PCR mix (μM)**
**Panel 1**					
*IL1-α*	1800587	GCAATAGACCTTATGACACCTAAC	GGTAGAGAAGAGAACAGTGGTATT	731	3.0
*IL1-β*	16944	AGTATATGTGGGACAAAGTGGAAG	CTCATCTGGCATTGATCTGGTT	389	1.4
*IL6*	1800795	AGCCTGTTAATCTGGTCACTGA	CTTGTGGAGAAGGAGTTCATAGC	498	3.2
*IL10*	1800872	TTCTATGTGCTGGAGATGGTGTA	GGGTGGGCTAAATATCCTCAAAG	385	1.4
*IL12*	11575934	GGTTAAGTGACTGGTGCCAAG	TCCTGTACTCAGAGTGATCTTACC	210	3.0
**Panel 2**					
*TLR2*	5743708	TGCTGCCATTCTCATTCTTCCTG	TCCTCAAATGACGGTACATCCA	282	1.4
*IL10*	1800896	ATCTGAAGAAGTCCTGATGTCACT	ACCATCTCCAGCACATAGAATGA	367	1.4
*TLR2*	4696480	CATAGTTGTCACAGTCCCTTGG	TGGCATGATCTCGGCTCAC	346	4.0
*IL6*	1800797	GGTGAAGAGACTCAGTGGCAAT	AGTGACCAGATTAACAGGCTAGAAT	444	2.0
*TLR4*	4986791	CCGATTAGCATACTTAGACTACTACCT	AGACTGGACAAGCCATTGAAGAT	555	3.2
**Panel 3**					
*IL12*	401502	ACCTCTGTATGACATTGAGTAAGC	GAACCAACGGGACCACCAT	285	1.0
*TLR2*	121917864	CTGTGCTCTGTTCCTGCTGAT	GAAATGGGAGAAGTCCAGTTCATAC	382	1.0
*TLR9*	187084	ATTCATTCATTCAGCCTTCACTCAG	CCATCCAGCCTTCTTACAAACCT	324	1.0
*CD14*	2569190	GGCTTCCAGGCTTCACACT	AGGACACTGCCAGGAGACA	236	1.0
*TLR4*	4986790	TCCCTGAACCCTATGAACTTTATCC	AGGCTTGGTAGATCAACTTCTGAA	494	1.0
*CD14*	2569191	AAGGAAGGGATGGGACTATGTT	CAGGAATCTGAGGCAAGAGAAT	347	1.0

**Table 3:** Extension probes used for multiplex primer extension

**Gene**	**Positionchange**	**Variation**	**Extension probe** **(5’→3’)**	**Probe size (bp)**	**PCR mix (μM)**
**Panel 1**					
*IL1-α*	−889	C>T	TTAATAATAGTAACCAGGCAACA	23	2.0
*IL1-β*	−511	A>G	TTTTTTTTTTGGTGCTGTTCTCTGCCTC	28	1.2
*IL6*	−174	C>G	TTTTTTTTTTTTTTTCCCCTAGTTGTGTCTTGC	33	2.0
*IL10*	−592	A>C	TTTTTTTTTTTTTTTTTTTTTCCTGTGACCCCGCCTGT	38	1.2
*IL12*	+705	A>G	TTTTTTTTTTTTTTTTTTTTTTTTTTCCAGCTCCGACGACGGC	43	2.0
**Panel 2**					
*TLR2*	+2259	A>G	TTTTTTTGCGCTTCTGCAAGCTGC	24	0.8
*IL10*	−1082	A>G	TTTTTTTTCACTACTAAGGCTTCTTTGGGA	30	0.8
*TLR2*	−16933	A>T	TTTTTTTTTTTTTTTTTATTGAAGGGCTGCATCTGG	36	3.2
*IL6*	−596	A>G	TTTTTTTTTTTTTTTTTTTTTGTAACTGCACGAAATTTGAGG	42	0.8
*TLR4*	+1196	C>T	TTTTTTTTTTTTTTTTTTTTTTTTGTTCTCAAAGTGATTTTGGGACAA	48	3.2
**Panel 3**					
*IL12*	+1196	C>G	TTTTCAGGTGGCAAGGCCCC	20	1.0
*TLR2*	+2029	C>T	TTTTTCCCTTCAAGTTGTGTCTTCATAAGC	30	1.4
*TLR9*	−1486	C>T	TTTTTTTTTTTTTTCAGATAAAAGATCACTGCCCT	35	1.0
*CD14*	−260	A>G	TTTTTTTTTTTTTTTTTTTTGGATGTTTCAGGGAGGGGGG	40	1.0
*TLR4*	+896	A>G	TTTTTTTTTTTTTTTTTTTAGCATACTTAGACTACTACCTCGATG	45	1.0
*CD14*	−159	C>T	TTTTTTTTTTTTTTTTTTTTTTTGAGGATTAATTAGTAACTCACCAGTTT	50	2.0

### Multiplex PCR amplification

Multiplex PCRs were performed in 10 μl total volume with Type-it® Mutation detect PCR kit (Qiagen) protocol. Thermal cycler conditions were: initial denaturation step at 95°C for 5 min, then 35 cycles at 95°C for 30 s, 50°C (panel 1) or 51°C (panels 2 and 3) for 90 s and 72°C for 30 s, followed by final extension at 68°C for 10 min. PCR products were treated with a mix of 5 U Exonuclease I (*Exo*I, Fermentas) and 1 U of FastAP™ Thermosensitive Alkaline Phosphatase (*SAP*, Fermentas) at 37°C for 15 min, and enzyme inactivation at 85°C for 15 min. PCR quality was evaluated after electrophoresis in a 3% agarose gel.

### SNaPshot analysis

SNaPshot analysis was performed using a SNaPshot Multiplex Kit protocol (Applied Biosystems). Products were treated with 1 U of *SAP* at 37°C for 60 min and 75°C for 15 min, followed by a denaturation step at 95°C for 5 min. Detection was carried out using 0.5 μl of SNaPshot products mixed with 9 μl of HiDi™ formamide and 0.5 μl of GeneScan-120LIZ size standard (Applied Biosystems). Data were generated after capillary electrophoresis on an automated sequencer (ABI 3130 Genetic Analyzer, Applied Biosystems) with a 36 cm length capillary and POP-7™ polymer and analyzed with GeneMapper analysis software version 3.7 (Applied Biosystems).

## Results and discussion

The strategy of the present work included the design of a multiplex assay, in order to detect 16 SNPs among nine genes of the innate immune system. For all panels, the specificity of each amplicon was evaluated first in singleplex and after in multiplex PCR, followed by electrophoresis in an agarose gel. According to the amplicons quality, primer concentrations and annealing temperatures were adjusted in order to obtain bands with equal intensities (Table 2). SNPs detection also implicated the optimisation of the three multiplex primer extension (SNaPshot) panels. In order to achieve more comparable peak heights, we adjusted probe concentrations (Table 3).

**Figure 1:**
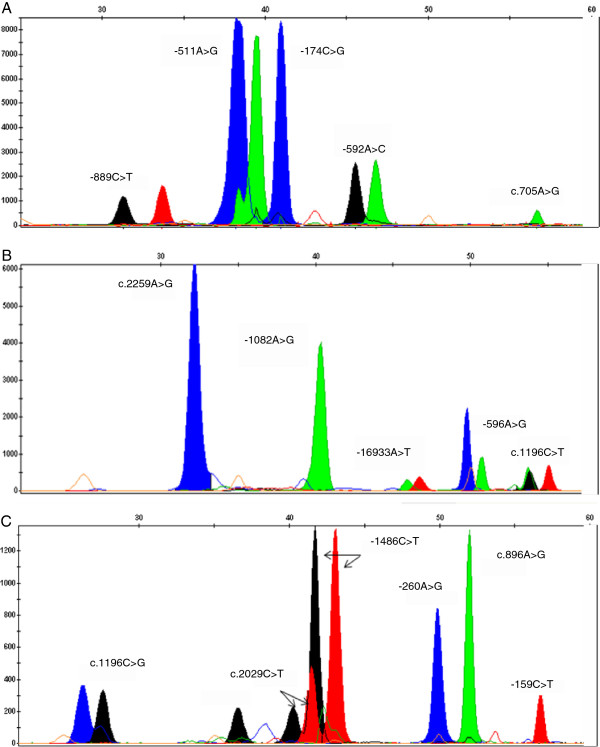
**Electropherograms obtained for the three multiplex primer extension panels.** Capillary electrophoresis of SNaPshot products was performed with an ABI 3130 Genetic Analyzer. (**A**) Panel 1 SNaPshot results obtained for *IL1-α*: rs1800587 (−889C>T), *IL1-β*: rs16944 (-511A>G), *IL6*: rs1800795 (−174C>G), *IL10*: rs1800872 (−592A>C) and *IL12*: rs11575934 (c.705A>G). (**B**) Panel 2 SNaPshot results for *TLR2*: rs5743708 (c.2259A>G), *IL10*: rs1800896 (−1082A>G), *TLR2*: rs4696480 (−16933A>T), *IL6*: rs1800797 (−596A>G) and *TLR4*: rs4986791 (c.1196C>T). (**C**) Panel 3 SNaPshot results for *IL12*: rs401502 (c.1196C>G), *TLR2*: rs121917864 (c.2029C>T), *TLR9*: rs187084 (−1486C>T), *CD14*: rs2569190 (−260A>G), *TLR4*: rs4986790 (c.896A>G) and *CD14*: rs2569191 (−159C>T). Nucleotides are represented by the following colours: A = green; C = black; G = blue; T = red.

Figure 1 represents the electropherograms obtained for each panel. Polymorphisms were identified on the basis of peak size and colour. As expected, minor shifts in the electrophoretic mobility were observed due to the incorporated base at the end of each probe and to the POP-7™ polymer. However, these shifts did not interfered with the analysis because poly-T tails increased probes spacing. Some samples evidenced one PCR product peak with more than one colour due to pull-ups, as illustrated in Figure 1A, *IL1-β* polymorphism -511A>G. The peak in blue produced a secondary peak in black or green. This problem is probably due to a bleed-through when an adjacent lane has very high peak heights. Nevertheless, it was resolved by reducing the concentration of the respective probe.

**Figure 2:**
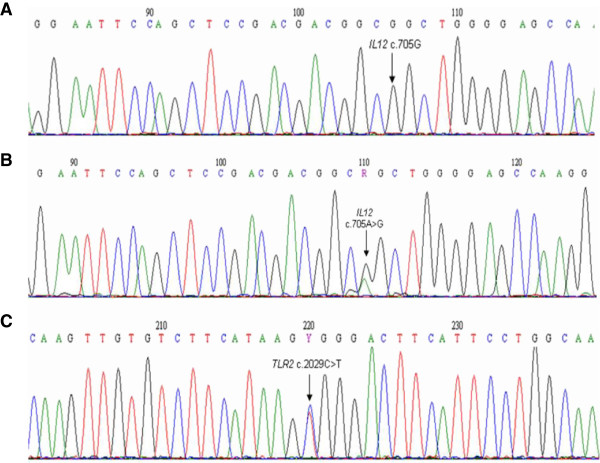
**Electropherograms obtained from Sanger DNA sequencing.** (**A**) and (**B**) corresponds to the DNA sequence flanking *IL12* gene at c.705A>G, being the genotypes indicated by the arrow – GG and AG – respectively. (**C**) Corresponds to the DNA sequence flanking *TLR2* at c.2029C>T. The arrow indicates the genotype CT.

The panels, here described, were evaluated in 100 DNA samples. From the 16 SNPs, we expected to identify 32 alleles and 48 different genotypes. However, we did not identify the G allele of the *IL12* c.705 A>G (the last SNP in panel 1) and its inherent genotypes (AG and GG). This missing allele is a failure of the minisequencing reaction, since the Sanger DNA sequencing (validation test) revealed the presence of the two genotypes in our sample (Figures 2A and 2B). Indeed, in a study carried out in the Brazilian population, the multiplex protocol, which was optimized for simultaneous detection of 12 autosomal SNPs, revealed that large amplicons (413 and 576 bp) had lower sensitivity to allele detection in multiplex minisequencing [15]. Moreover, two homozygous genotypes were not observed in *TLR2*, namely: AA for c.2259A>G, and TT for c.2029C>T (panel 2 and 3, respectively). According to the SNP database (dbSNP), these genotypes were not found in the 11 HapMap populations (phase 3), which includes the Europeans (CEU). In fact, the A allele (c.2259A>G) is rare – 0.052 in CEU – and was only detected in homozygosity in tuberculosis patients [16]; the T allele (c.2029C>T) is observed in Asian and African populations, but it seems to be absent in white populations [17,18]. Nevertheless, in the present work, the T allele was identified in heterozygosity in one individual, as illustrated in Figure 1C (c.2029C>T) and was confirmed by sequencing (Figure 2C). This finding is not surprising since our previous studies show African influence in São Miguel population [19,20].

In order to validate our assays, we selected 25 DNA samples for Sanger DNA sequencing. The results confirmed the 44 genotypes previously obtained by multiplex primer extension assay, as well as the two corresponding genotypes (AG and GG) of the missing allele *IL12* c.705G.

Considering that these nine candidate innate immune genes have been investigated in autoimmune and infectious diseases through association studies, the three panels here described may allow the detection of at least 15 risk gene variants in all populations. Panel 1, for example, contains 4 SNPs in interleukin genes, which are often related to autoinflammatory diseases [21]. On the other hand, panel 2 and 3 could be used for polymorphism genotyping (5 and 6 SNPs, respectively) in studies of infectious diseases associated with microbial pathogens, whose recognition by the innate immune system depends on Toll-like receptors [22].

## Conclusions

With this study, we were able to develop combined multiplex PCR amplifications and SNaPshot reactions that genotypes 15 SNPs among nine innate immune response genes. These multiplex panels can be useful to investigate candidate immune response genes, using a medium scale genotyping platform, rather than singleplex PCR-based techniques.

## Competing interests

The authors declare that they have no competing interests.

## Authors’ contributions

LME developed and performed the multiplex experiments and drafted the manuscript. SMB and MJB did the validation assays by Sanger DNA sequencing. LMV provided scientific orientation and revised the manuscript. All authors read and approved the final manuscript.
